# Factors Associated With Recurrence in Patients With Benign Paroxysmal Positional Vertigo Following Canal Repositioning Therapy

**DOI:** 10.7759/cureus.117036

**Published:** 2026-09-27

**Authors:** Navdeep Kaur, Rachna Dhingra, Sumit Prinja

**Affiliations:** 1 Department of Otorhinolaryngology, Guru Gobind Singh Medical College and Hospital, Faridkot, IND

**Keywords:** benign paroxysmal positional vertigo, diabetes mellitus, hypertension, recurrence, risk factors

## Abstract

Introduction: Benign paroxysmal positional vertigo (BPPV) is effectively managed with canal repositioning therapy; however, recurrence is common. Identifying clinical factors associated with recurrence may help improve follow-up and patient counselling. This study aimed to determine the recurrence of BPPV following canal repositioning therapy and to evaluate the demographic and clinical factors associated with recurrence.

Materials and methods: This prospective observational study included 100 patients with clinically diagnosed BPPV who underwent canal repositioning therapy in the Department of Otorhinolaryngology at Guru Gobind Singh Medical College and Hospital, Faridkot. Demographic characteristics, comorbidities, clinical presentation, affected side, and involved semicircular canal were recorded for all patients. Patients were followed up for recurrence, and potential predictors were evaluated using univariate logistic regression analysis.

Results: Recurrence occurred in 27 (27.0%) patients, whereas 73 (73.0%) remained recurrence-free during follow-up. Posterior canal involvement was predominant in both groups. Hypertension and diabetes mellitus were significantly associated with recurrence in univariate analysis, whereas sex, affected side, and involved semicircular canal were not significantly associated with recurrence. Among patients who developed recurrence, 12 (44.4%) experienced recurrence within one month, 10 (37.0%) between one and three months, and 5 (18.5%) between three and six months.

Conclusion: Recurrence following canal repositioning therapy was significantly associated with hypertension and diabetes mellitus in the present study. Recognition of these factors may support individualized counselling and closer follow-up of patients at greater risk of recurrence.

## Introduction

Vertigo is a common and distressing complaint in otorhinolaryngology that may result in functional limitations and an increased risk of falls. Among peripheral vestibular disorders, benign paroxysmal positional vertigo (BPPV) is frequently encountered [1]. It is characterized by brief episodes of vertigo and positional nystagmus, which are precipitated by changes in head position relative to gravity, such as during positional changes [2]. BPPV results from the displacement of otoconia from the utricular macula into a semicircular canal, producing abnormal stimulation during head movement [3]. Canalithiasis, in which otoconia remain free within the canal, and cupulolithiasis, in which particles adhere to the cupula, are the main mechanisms [3].

The posterior semicircular canal is most commonly involved because of its anatomical orientation and gravitational dependence [4]. The Dix-Hallpike test is the established diagnostic maneuver for posterior canal BPPV [5], while canal repositioning procedures, particularly the Epley maneuver, are widely used to restore displaced otoconia to the utricle [6]. These procedures provide high rates of symptomatic relief; however, successful initial treatment does not eliminate the risk of recurrence. Published studies have reported recurrence rates ranging from approximately 13.7% to more than 48% with follow-up <1 year and from 13.3% to 65% for studies with follow-up ≥2 years, depending on the population and duration of follow-up [7]. Recurrence can result in repeated episodes of vertigo, anxiety, falls, functional impairment, and healthcare utilization [7,8].

The mechanisms underlying BPPV recurrence are multifactorial and may involve persistent or recurrent otoconial instability, age-related degeneration of the vestibular apparatus, and systemic or metabolic factors [8]. Increasing age has been associated with greater susceptibility to otoconial detachment and recurrent BPPV [8]. Hypertension may contribute to recurrence through vascular injury and impaired labyrinthine perfusion, whereas diabetes mellitus may affect otolith function and vestibular homeostasis [7,9]. However, evidence regarding the influence of sex and other clinical characteristics on recurrence remains inconsistent [9]. Identifying factors associated with recurrence may help clinicians provide individualized counselling and determine the need for closer follow-up. Despite the established effectiveness of canal repositioning therapy, recurrence remains clinically relevant, and the determinants of recurrence are not yet consistently established. Therefore, this hospital-based prospective observational study aimed to determine the recurrence of BPPV following canal repositioning therapy and to evaluate the associations of age, sex, hypertension, and diabetes mellitus with recurrence.

## Materials and methods

Study design and setting

This hospital-based prospective observational study was conducted at the Department of Otorhinolaryngology, Guru Gobind Singh Medical College and Hospital, Faridkot, Punjab, India. The study was conducted over a period of 18 months, between July 2024 and December 2025. The study protocol was reviewed and approved by the Institutional Ethics Committee (GGS/IEC/09, dated May 23, 2024). Before enrollment, all eligible participants were informed of the purpose and procedures of the study in their vernacular language. Written informed consent was obtained from all participants before inclusion. Participation was voluntary, and the participants were informed that they could withdraw from the study at any time without affecting their clinical care. Patient-related information was kept confidential throughout the study period.

Study population

Patients aged 18-70 years of either sex with a clinical diagnosis of BPPV and a positive Dix-Hallpike test result were considered eligible [5]. Patients with central causes of positional vertigo were excluded from the study. Eligibility criteria were established to obtain a relatively homogeneous population with peripheral positional vertigo suitable for canal repositioning therapy.

Eligibility criteria

Patients were included if they were aged 18-70 years, of either sex, had a clinical diagnosis of BPPV, and demonstrated a positive Dix-Hallpike test after exclusion of central causes of vertigo [5]. Patients with bilateral positive Dix-Hallpike test results were excluded. Other exclusion criteria included central causes of vertigo, such as brainstem transient ischemic attack or infarction, multiple sclerosis, posterior fossa tumors, and temporal lobe epilepsy; a history of head injury or trauma; previous ear disease or ear surgery; previous neurosurgical procedures; known cervical spondylitis; and use of anti-vertigo medications.

Sample size and sampling technique

The statistical document reports that a priori sample size estimation was performed using G*Power software, version 3.1.9.7 (Heinrich Heine University Düsseldorf, Düsseldorf, Germany), based on a one-sample proportion model. A previously reported BPPV recurrence rate of 29.4% was used for estimation, with 80% statistical power, a 95% confidence level (α = 0.05), and an allowable margin of error of 10% [10]. The estimated minimum sample size was 80. Considering the potential loss to follow-up (approximately 20%), incomplete records, or missing data, the target sample was increased to 96, and 100 patients were ultimately targeted and included to improve robustness.

Clinical assessment and data collection

All participants underwent detailed history taking, clinical examination, and complete otorhinolaryngological examination using a structured study proforma. Demographic characteristics, including age and sex, were recorded. Information on relevant comorbidities, particularly hypertension and diabetes mellitus, was also documented. The clinical characteristics of BPPV, including the affected side, involved semicircular canal, presenting symptoms, and positional triggers, were recorded.

Diagnosis of BPPV

BPPV was clinically diagnosed using the Dix-Hallpike test [5]. The patient was initially seated upright on an examination couch. The head was rotated 45° toward the side being tested to appropriately align the posterior semicircular canal. The patient was then rapidly brought into the supine position with the head extended approximately 30° below the horizontal edge of the couch while maintaining a 45° head rotation. Patients were informed beforehand that the maneuver could provoke vertigo and were instructed to keep their eyes open so that nystagmus could be observed. A positive test was based on the reproduction of positional vertigo accompanied by characteristic positional nystagmus. Patients with a negative Dix-Hallpike test result were excluded. Patients with a positive test result were subsequently treated with canal repositioning.

Canal repositioning therapy

Patients with BPPV underwent the Epley canal repositioning maneuver [6]. The patient was initially seated on the examination couch with the body aligned longitudinally and facing away from the examiner. The patient was rapidly positioned in the supine position with the head turned 45° toward the affected side and slightly extended over the edge of the couch, corresponding to the initial position used during the Dix-Hallpike test. The head was then slowly rotated 90° toward the unaffected side. The patient's body was rotated in the same direction while maintaining the appropriate head position. The head was further rotated by another 90° so that the patient faced slightly downward. The patient was then assisted in moving the legs over the side of the couch and returning to the sitting position. Finally, the patient was brought upright with the head maintained at approximately 45° toward the unaffected side and slightly flexed forward by approximately 20°. If symptoms and positional nystagmus persisted after the initial maneuver, the canal repositioning maneuver was repeated as clinically indicated. The number of maneuvers required for symptom resolution was recorded.

Assessment of recurrence

Recurrence was assessed after successful canal repositioning therapy. Recurrence was defined as the reappearance of positional vertigo and nystagmus after at least one month of symptom-free status following effective canal repositioning therapy. Participants were scheduled for follow-up in the Otorhinolaryngology Outpatient Department at one, three, and six months after treatment to assess their clinical status and identify recurrence. Participants were instructed to report to the department earlier if they developed recurrent vertigo before the scheduled follow-up visit. Recurrence was recorded as a binary outcome (recurrence versus no recurrence). The time interval between canal repositioning therapy and recurrence was recorded and categorized according to the study data.

Variables assessed

The primary outcome was the recurrence of BPPV following canal repositioning therapy. The principal explanatory variables specified in the study objectives were age, sex, hypertension, and diabetes mellitus. Additional demographic and clinical variables recorded included residence, occupation, affected ear, involved semicircular canal, presenting symptoms such as nausea, imbalance or unsteadiness, vomiting, headache, and tinnitus, and positional triggers, including turning in bed, getting up from bed, bending forward, and looking upward. These additional variables were subsequently examined as potential factors associated with recurrence.

Statistical analysis

Data were entered into Microsoft Excel (Microsoft Corporation, Redmond, WA, USA) and subsequently analyzed using IBM SPSS Statistics, version 25.0 (IBM Corporation, Armonk, NY, USA). Continuous variables were assessed for distribution using the Shapiro-Wilk test. Normally distributed continuous variables were summarized as mean ± standard deviation, whereas categorical variables were summarized as frequencies and percentages. Appropriate parametric or non-parametric tests were selected according to the distribution and nature of the variables. Categorical variables were compared using the chi-square test or Fisher's exact test, as appropriate. For comparisons involving small expected cell frequencies, Fisher's exact test or the Fisher-Freeman-Halton test was used. Univariate logistic regression analysis was performed to examine the association between individual candidate factors and recurrence. Odds ratios (ORs) with corresponding 95% confidence intervals (CIs) were calculated for each candidate factor. Candidate factors included age, sex, residence, hypertension, diabetes mellitus, affected side, clinical symptoms, and positional triggers. A two-sided p-value <0.05 was considered statistically significant.

## Results

A total of 100 patients with BPPV were included in this study. Of these, 73 (73.0%) did not develop recurrence during follow-up, whereas 27 (27.0%) experienced recurrence following canal repositioning therapy. The mean age was 49.08 ± 14.04 years in the non-recurrence group and 49.63 ± 12.15 years in the recurrence group, with no statistically significant difference between the groups (p = 0.920). Female patients constituted 44 (60.3%) and 17 (63.0%) patients in the non-recurrence and recurrence groups, respectively, while male patients constituted 29 (39.7%) and 10 (37.0%) patients, respectively. Sex was not significantly associated with recurrence (p = 0.989). Similarly, residence and occupation did not differ significantly between the two groups (Table 1).

**Table 1:** Comparison of sociodemographic characteristics between patients with and without recurrence. Data are presented as mean ± standard deviation for age and as frequency (percentage) for categorical variables. Age was compared using the Mann-Whitney U test, and categorical variables were compared using the chi-square test or Fisher-Freeman-Halton test, as appropriate. A two-sided p-value <0.05 was considered statistically significant. Effect sizes are reported as r for the Mann-Whitney U test, φ (phi) for 2 × 2 categorical comparisons, and Cramér’s V for comparisons involving more than two categories.

Variable		No recurrence (n = 73)	Recurrence (n = 27)	Test statistic	p-Value	Effect size
Age, years	Mean ± SD	49.08 ± 14.04	49.63 ± 12.15	U = 999.0	0.920	r = 0.010
Sex, n (%)	Female	44 (60.3%)	17 (63.0%)	χ² = 0.000	0.989	φ = 0.001
	Male	29 (39.7%)	10 (37.0%)			
Residence, n (%)	Rural	45 (61.6%)	14 (51.9%)	χ² = 0.429	0.513	φ = 0.065
	Urban	28 (38.4%)	13 (48.1%)			
Occupation, n (%)	Housewife	34 (46.6%)	11 (40.7%)	Fisher-Freeman-Halton	0.755	Cramér’s V = 0.156
	Farmer/laborer	17 (23.3%)	10 (37.0%)			
	Service/employee	11 (15.1%)	4 (14.8%)			
	Retired/others	6 (8.2%)	1 (3.7%)			
	Student	5 (6.8%)	1 (3.7%)			

The distribution of comorbidities differed significantly according to the recurrence status (p < 0.001). Among patients without recurrence, 41 (56.2%) had no comorbidity, compared with only 1 (3.7%) patient among those with recurrence. Hypertension alone was present in 16 (21.9%) patients without recurrence and 8 (29.6%) patients with recurrence, whereas diabetes mellitus alone was present in 11 (15.1%) and 4 (14.8%) patients, respectively. The coexistence of hypertension and diabetes mellitus was observed in 5 (6.8%) patients without recurrence and 14 (51.9%) patients with recurrence (Table 2).

**Table 2:** Comparison of comorbidities and clinical characteristics of benign paroxysmal positional vertigo between patients with and without recurrence. Data are presented as frequency (percentage). Categorical variables were compared using the chi-square test or Fisher-Freeman-Halton test, as appropriate. Effect sizes are reported as φ (phi) for 2 × 2 comparisons and Cramér’s V for comparisons involving more than two categories. *A two-sided p-value <0.05 was considered statistically significant.

Variable		No recurrence (n = 73)	Recurrence (n = 27)	Test statistic	p-Value	Effect size
Comorbidity profile, n (%)	No comorbidity	41 (56.2%)	1 (3.7%)	χ² = 34.414	<0.001*	Cramér’s V = 0.587
	Hypertension only	16 (21.9%)	8 (29.6%)			
	Diabetes only	11 (15.1%)	4 (14.8%)			
	Hypertension + diabetes	5 (6.8%)	14 (51.9%)			
Affected side, n (%)	Left ear	33 (45.2%)	11 (40.7%)	χ² = 0.030	0.863	φ = 0.017
	Right ear	40 (54.8%)	16 (59.3%)			
Involved canal, n (%)	Posterior canal	63 (86.3%)	24 (88.9%)	Fisher-Freeman-Halton	1.000	Cramér’s V = 0.087
	Horizontal canal	8 (11.0%)	3 (11.1%)			
	Anterior canal	2 (2.7%)	0 (0.0%)			

The affected sides were comparable between the groups. Right-sided BPPV was observed in 40 (54.8%) patients without recurrence and 16 (59.3%) patients with recurrence, whereas left-sided involvement was observed in 33 (45.2%) and 11 (40.7%) patients, respectively (p = 0.863). Posterior canal involvement was predominant in both groups, occurring in 63 (86.3%) patients without recurrence and 24 (88.9%) patients with recurrence. No significant association was observed between the involved semicircular canal and recurrence (p = 1.000) (Table 2).

Positional vertigo was observed in all 100 (100.0%) patients. Nausea was reported by 68 (68.0%) patients, imbalance or unsteadiness by 42 (42.0%), vomiting by 24 (24.0%), headache by 18 (18.0%), and tinnitus by 9 (9.0%) patients. Turning in bed was the most frequently reported provoking factor, occurring in 53 (53.0%) patients, followed by getting up from bed in 27 (27.0%), bending forward in 12 (12.0%), and looking upward or neck extension in 8 (8.0%) patients. When clinical characteristics were compared according to recurrence status, hypertension and diabetes mellitus were significantly associated with recurrence. Hypertension was present in 22 (81.5%) patients with recurrence and 21 (28.8%) without recurrence (OR = 10.69, 95% CI: 3.57-31.97; p < 0.001). Diabetes mellitus was present in 18 (66.7%) patients with recurrence and 16 (21.9%) without recurrence (OR = 7.13, 95% CI: 2.69-18.86; p < 0.001). Vomiting, tinnitus, imbalance, headache, nausea, and individual positional triggers were not significantly associated with recurrence (Table 3).

**Table 3:** Association of individual comorbidities, presenting symptoms, and positional triggers with recurrence. Data are presented as frequency (percentage). Associations between categorical variables and recurrence were assessed using appropriate categorical tests. ORs with 95% CIs are presented as measures of association, and φ (phi) is reported as the effect size for 2 × 2 comparisons. *A two-sided p-value <0.05 was considered statistically significant. OR: odds ratio; CI: confidence interval.

Variable	No recurrence, n (%)	Recurrence, n (%)	OR (95% CI)	p-Value	Effect size
Hypertension	21 (28.8%)	22 (81.5%)	10.69 (3.57-31.97)	<0.001*	φ = 0.468
Diabetes	16 (21.9%)	18 (66.7%)	7.13 (2.69-18.86)	<0.001*	φ = 0.419
Nausea	50 (68.5%)	18 (66.7%)	0.92 (0.36-2.36)	1.000	φ = 0.017
Imbalance/unsteadiness	28 (38.4%)	14 (51.9%)	1.73 (0.71-4.22)	0.259	φ = 0.121
Vomiting	14 (19.2%)	10 (37.0%)	2.48 (0.94-6.57)	0.072	φ = 0.186
Headache	13 (17.8%)	5 (18.5%)	1.05 (0.34-3.28)	1.000	φ = 0.008
Tinnitus	4 (5.5%)	5 (18.5%)	3.92 (0.97-15.89)	0.057	φ = 0.202
Turning in bed	41 (56.2%)	12 (44.4%)	0.62 (0.26-1.52)	0.369	φ = 0.104
Getting up from bed	16 (21.9%)	11 (40.7%)	2.45 (0.95-6.31)	0.077	φ = 0.188
Bending forward	6 (8.2%)	6 (22.2%)	3.19 (0.93-10.95)	0.081	φ = 0.191
Looking upward/extension	6 (8.2%)	2 (7.4%)	0.89 (0.17-4.72)	1.000	φ = 0.013

Following canal repositioning therapy, 73 (73.0%) patients achieved complete symptomatic relief after a single maneuver. A second maneuver was required in 20 (20.0%) patients, whereas 7 (7.0%) required three maneuvers. Overall, 27 (27.0%) patients developed recurrent BPPV during follow-up. Among patients who developed recurrence, 12 (44.4%) experienced recurrence within one month, 10 (37.0%) experienced recurrence between one and three months, and 5 (18.5%) experienced recurrence between three and six months. Thus, most recurrence events occurred during the earlier period after canal repositioning therapy (Figure 1).

**Figure 1:**
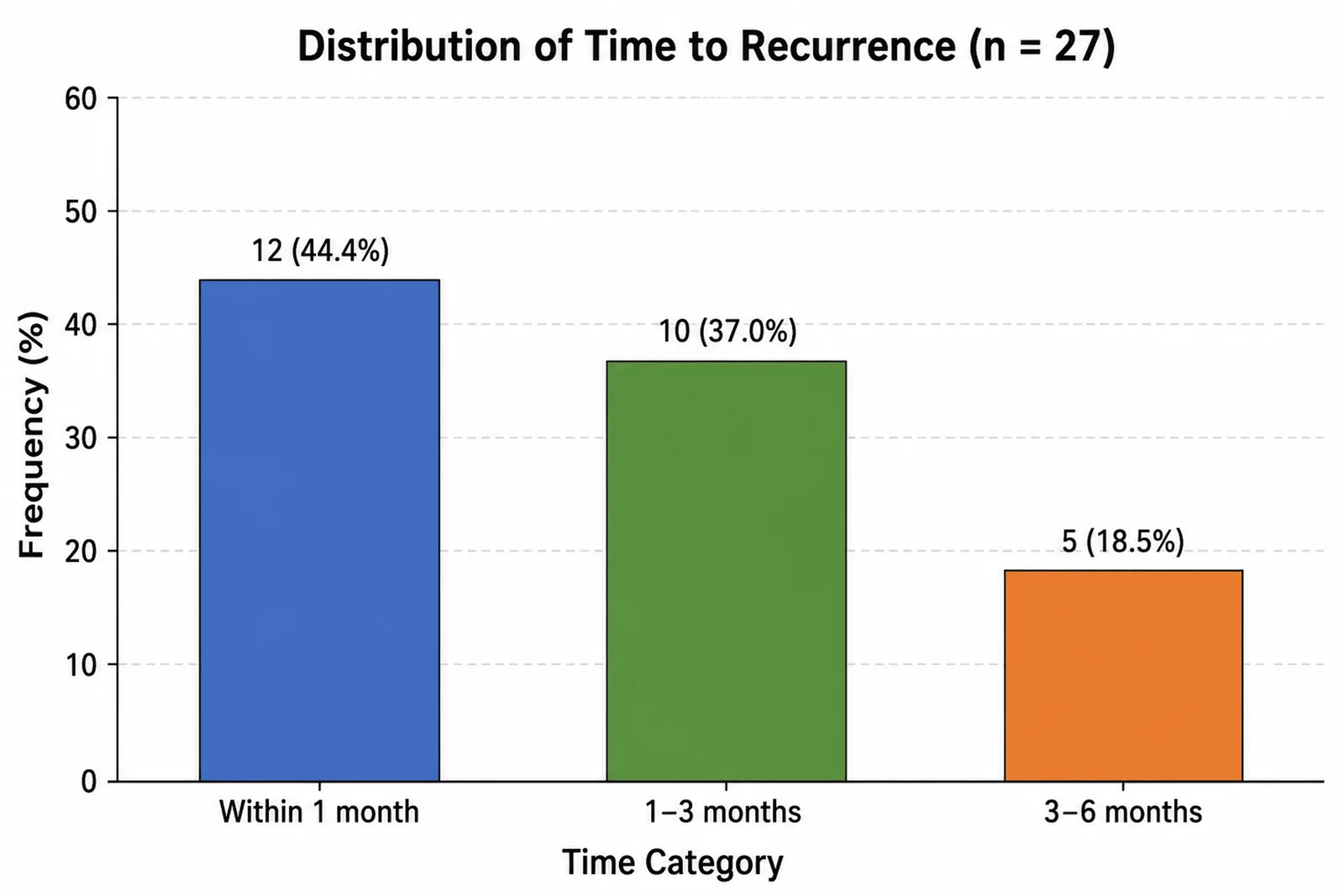
Distribution of patients according to the time interval between canal repositioning therapy and recurrence. Data are presented as frequency (percentage) among patients who developed recurrence (n = 27).

Screening analysis identified hypertension and diabetes mellitus as significant predictors of recurrence. Hypertension was associated with approximately 10-fold higher odds of recurrence (OR 10.686, 95% CI 3.571-31.973; p < 0.001), whereas diabetes mellitus was associated with more than seven-fold higher odds (OR 7.125, 95% CI 2.691-18.862; p < 0.001). Age, male sex, urban residence, right-sided BPPV, imbalance, vomiting, tinnitus, nausea, headache, and positional triggers were not statistically significant in the screening analysis (Table 4).

**Table 4:** Univariate logistic regression analysis of potential factors associated with recurrence. Each candidate factor was evaluated in a univariate logistic regression model with recurrence as the outcome. *A two-sided p-value <0.05 was considered statistically significant. OR: odds ratio; CI: confidence interval; BPPV: benign paroxysmal positional vertigo.

Candidate predictor	Association measure	95% CI	p-Value	Interpretation
Age, per year	OR = 1.003	0.970-1.037	0.856	No association
Male sex	OR = 0.892	0.359-2.219	1.000	No association
Urban residence	OR = 1.492	0.613-3.635	0.493	No association
Hypertension	OR = 10.686	3.571-31.973	<0.001*	Strong predictor candidate
Diabetes	OR = 7.125	2.691-18.862	<0.001*	Strong predictor candidate
Right-sided BPPV	OR = 1.200	0.490-2.938	0.821	No association
Imbalance/unsteadiness	OR = 1.731	0.711-4.215	0.259	Weak-moderate association
Vomiting	OR = 2.479	0.936-6.569	0.072	Potential predictor
Tinnitus	OR = 3.920	0.967-15.892	0.057	Potential predictor
Getting up from bed	OR = 2.449	0.950-6.314	0.077	Potential predictor
Bending forward	OR = 3.190	0.930-10.950	0.081	Potential predictor
Turning in bed	OR = 0.624	0.257-1.519	0.369	No association
Nausea	OR = 0.920	0.359-2.356	1.000	No association
Headache	OR = 1.049	0.335-3.284	1.000	No association
Looking upward	OR = 0.893	0.169-4.722	1.000	No association

## Discussion

The present study found that recurrence after canal repositioning therapy occurred in a substantial proportion of patients with BPPV, highlighting that successful initial treatment does not necessarily prevent subsequent episodes. Previous reviews have reported considerable variations in recurrence rates depending on the duration of follow-up and study population, supporting the clinical importance of continued observation after apparently successful repositioning therapy [7,8]. Because multiple demographic and clinical variables were examined, the possibility of chance findings due to multiple comparisons cannot be excluded, and borderline associations should therefore be interpreted cautiously.

Age was not significantly associated with recurrence when analyzed as a continuous variable in the univariate analysis. Nevertheless, the potential relationship between increasing age and BPPV recurrence has been described in previous studies and recent meta-analytic evidence. Age-related degeneration of utricular otoconia, alterations in calcium homeostasis, and progressive changes in the vestibular apparatus may contribute to repeated otoconial detachment [11]. Reduced physical activity and mobility in older adults may further influence normal otolith clearance. Previous studies have reported an association between advanced age and BPPV recurrence, although findings have not been consistent across studies [8,12,13]. The lack of a significant association with age in the present study may reflect differences in study populations, sample size, follow-up duration, or the influence of other age-related comorbidities.

Hypertension was significantly associated with recurrence in the present study. Vascular abnormalities associated with chronic hypertension may influence the microcirculation of the inner ear and potentially impair vestibular homeostasis [14]. Hypertension may coexist with other vascular and metabolic abnormalities that could affect otoconial integrity. This finding is consistent with previous meta-analyses that identified hypertension as a risk factor for BPPV recurrence [8,15]. However, the evidence is not entirely uniform; another meta-analysis evaluating risk factors for BPPV more broadly did not find a significant association between hypertension and the initial occurrence of BPPV [15]. This distinction suggests that hypertension may be more relevant to the persistence or recurrence than to the initial development of the disorder.

Diabetes mellitus was significantly associated with recurrence in the present study. Chronic hyperglycemia may cause microvascular dysfunction and metabolic alterations within the inner ear, potentially affecting the utricle and otoconia [16]. Diabetes may also be associated with impaired calcium metabolism and vascular changes, providing plausible mechanisms for repeated otoconial displacements. These findings are supported by previous systematic reviews and meta-analyses reporting an association between diabetes and recurrent BPPV [7]. Nevertheless, studies investigating the occurrence of BPPV have produced less consistent results, suggesting that the relationship may be influenced by disease duration, glycemic control, and accompanying vascular or metabolic conditions [17].

The coexistence of hypertension and diabetes was particularly prominent in patients who experienced recurrence. This observation may indicate that the cumulative vascular and metabolic burden, rather than a single comorbidity in isolation, contributes to the susceptibility to recurrent otoconial dysfunction. This finding is clinically relevant because both conditions are common and potentially modifiable, although the observational nature of the present study does not establish a causal relationship.

No significant association was observed between sex and recurrence after adjustment. Although some previous studies and meta-analyses have reported a higher recurrence risk among women, particularly in association with postmenopausal bone and calcium metabolism, the literature remains inconsistent [7]. Similarly, affected side and semicircular canal involvement were not associated with recurrence in this study. This is consistent with evidence indicating that the side and type of the involved canal are not consistently predictive of recurrence [18].

Most recurrences occurred relatively early after treatment, emphasizing the importance of patient education regarding the possibility of relapse and the recognition of recurrent positional symptoms. Clinically, older patients and those with hypertension or diabetes may benefit from more individualized counselling and closer follow-up [19]. Management should nevertheless remain focused on prompt repeat canalith repositioning when recurrence occurs, rather than assuming that initial treatment failure has occurred. Attention to metabolic and vascular comorbidities may also be part of comprehensive long-term management [19,20].

The clinical implication of these findings is that recurrence risk assessment can be incorporated into routine BPPV counselling. Identifying older patients and those with hypertension or diabetes may help clinicians set realistic expectations regarding recurrence and emphasize early reassessment when positional symptoms return. However, these factors should complement, rather than replace, clinical diagnosis and positional testing.

This study has several limitations. The relatively small, single-center sample may limit generalizability. The observational design permits the identification of associations but cannot establish causality. Important potential determinants, such as vitamin D status, osteoporosis, migraine, lipid abnormalities, duration and control of diabetes or hypertension, and bone mineral status, have not been comprehensively evaluated. In addition, recurrence may be influenced by the duration and completeness of follow-up, as well as by patient recognition and reporting of subsequent episodes. Larger multicenter prospective studies incorporating metabolic, vascular, and vestibular variables are warranted to develop more robust recurrence-prediction models.

## Conclusions

Recurrence following canal repositioning therapy remains clinically relevant in patients with BPPV. In the present study, hypertension and diabetes mellitus were significantly associated with recurrence in the univariate analysis, whereas age, sex, affected side, and semicircular canal involvement were not significantly associated with recurrence. These findings suggest that consideration of relevant patient-related factors may assist clinical counselling and follow-up. Patients with hypertension or diabetes mellitus may warrant closer monitoring and timely reassessment if positional symptoms recur, while canal repositioning remains the mainstay of management. Given the observational design and modest number of recurrence events, these findings should be interpreted as preliminary and warrant confirmation in larger, multicenter studies using multivariable analyses to account for potential confounding.
